# Physicochemical Changes in Root-Canal Sealers under Thermal Challenge: A Comparative Analysis of Calcium Silicate- and Epoxy-Resin-Based Sealers

**DOI:** 10.3390/ma17081932

**Published:** 2024-04-22

**Authors:** Hye-In Kim, Young-Eun Jang, Yemi Kim, Bom Sahn Kim

**Affiliations:** 1 Department of Conservative Dentistry, College of Medicine, Ewha Womans University, Seoul 07986, Republic of Korea; hyeineee@ewhain.net (H.-I.K.); yemis@ewha.ac.kr (Y.K.); 2 Department of Nuclear Medicine, College of Medicine, Ewha Womans University, Seoul 07986, Republic of Korea

**Keywords:** calcium silicate-based sealers, epoxy-resin sealer, warm obturation technique, heat application, physicochemical properties

## Abstract

Introduction: We compared the effects of heat on the physicochemical properties of recently developed calcium silicate-based sealers (CSBSs), including BioRoot Flow, BioRoot RCS, and AH Plus Bioceramic sealer, with those of the epoxy-resin-based sealer (ERBS) AH Plus. Methods: The flow, film thickness, setting time, and solubility of sealers were evaluated at 37 °C and 100 °C using ISO 6876/2012. Furthermore, pH and calcium ion release were evaluated at these temperatures. In addition, the mass change in sealers at a high temperature was assessed via thermogravimetric analysis. Then, the chemical composition and components of the sealers were analyzed using a scanning electron microscope and Fourier-transform infrared spectroscopy (FTIR). Results: BioRoot Flow, AH Plus Bioceramic, and AH Plus complied with ISO standards in terms of flow and film thickness, both before and after heat application. However, BioRoot RCS exhibited significantly increased film thickness at 100 °C. The setting times of all sealers were significantly reduced at 100 °C. The solubility of CSBS was >3%, exceeding the ISO 6876/2012 standard, both before and after heat exposure. Conversely, the solubility of AH Plus complied with the standard, regardless of the thermal condition. For 4 weeks, CSBS showed a significantly higher pH than AH Plus at both 37 °C and 100 °C. After heat treatment, calcium release decreased in Bioroot RCS and BioRoot Flow, while AH Plus showed no significant differences before and after treatment. However, CSBS consistently exhibited significantly higher calcium release than AH Plus at both temperatures. An FTIR analysis revealed that the chemical composition of the sealers did not change at the high temperature, whereas a thermogravimetric analysis demonstrated a >5% weight reduction in CSBS and a 0.005% weight reduction in AH Plus at 100 °C. Conclusions: BioRoot Flow, AH Plus Bioceramic, and AH Plus possess favorable physicochemical properties, which make them suitable for application under thermal conditions. At a high temperature, BioRoot RCS did not exhibit changes in its chemical composition. However, its film thickness was increased, and pH and solubility were reduced. Therefore, caution is needed when it is applied at high temperatures, such as during the warm obturation technique.

## 1. Introduction

The main purpose of root-canal treatment is to eliminate bacteria through the cleaning and shaping of the root canal, ensuring complete obturation and preventing reinfection [1]. Grossman et al. [2]. suggested that the ideal root-canal sealer should exhibit sufficient flow for penetration, an appropriate setting time, insolubility in the oral environment, biocompatibility, and safety. For several years, AH Plus (Dentsply Sirona, Konstanz, Germany), an epoxy-resin-based sealer (ERBS), has been used in warm and lateral obturation techniques. It possesses the advantages of long-term dimensional stability and high bond strength [3]. However, it lacks bioactive properties [4] and loses its antibacterial effects after the completion of setting [5]. To address these disadvantages, calcium silicate-based sealers (CSBS) have been developed recently, demonstrating several advantages, such as high alkalinity, calcium ion release, apatite formation, and mineralization [6,7].

CSBS is a hydraulic material that sets in the presence of moisture [8]. The hydration reaction, which produces calcium silicate hydrate during setting, is responsible for bioactivity, antibacterial properties, and tissue mineralization [9]. BioRoot RCS (Septodont, Saint-Maur-des-Fossés, France) is a commonly used CSBS that induces periodontal regeneration due to its antibacterial, anti-inflammatory, and bioactive properties [10].

The recently introduced AH Plus Bioceramic (Dentsply Sirona) has a short setting time and does not cause tooth discoloration [11]. Furthermore, according to the manufacturer, the most recently introduced pre-mixed CSBS, BioRoot Flow (Septodont), forms a mineral-rich hybrid layer between the sealer and dentin, has superior adhesion to gutta-percha and dentin, and can be used during the warm obturation technique [12].

When filling root canals, the warm obturation technique provides more effective filling in complex root canals than the lateral compaction technique by increasing gutta-percha density [13] and creating occlusions suitable for irregular root canals [14,15] However, excessive heat can alter the physiochemical properties of the root-canal sealer. A previous study demonstrated irreversible chemical changes in the ERBS AH Plus at high temperatures, including decreased setting time and flow and increased film thickness [16,17,18]. Conversely, other studies have observed a stable physiochemical structure and properties of AH Plus at high temperatures [19]. At high temperatures, the CSBS BioRoot RCS exhibits altered flow and film thickness due to moisture evaporation [20,21], whereas AH Plus Bioceramic maintained physiochemical properties [22], indicating varying effects of heat on sealers. To the best of our knowledge, few studies have evaluated the physiochemical, calcium ion release, and surface properties of BioRoot Flow after heat treatment.

The present study investigated the impact of heat on the physical, chemical, and surface properties of recently developed CSBSs, including BioRoot Flow, BioRoot RCS, and AH Plus Bioceramic sealer. These effects were evaluated using international standards and were compared to the properties of AH Plus following heat treatment. The null hypothesis stated that heat treatment does not influence the physiochemical properties of CSBS and AH Plus.

## 2. Materials and Methods

### 2.1. Heat Application on Sealer

**Figure 1:**
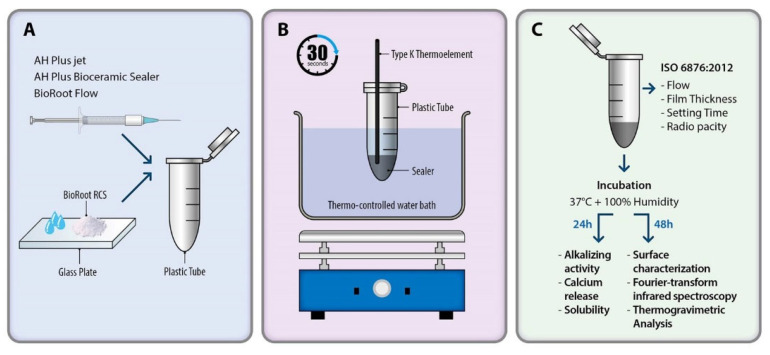
Schematic representation of experimental temperature settings. (**A**) Distributing sealers in plastic tubes. (**B**) The temperatures of the sealers were adjusted to 37 °C and 100 °C, maintained for 30 s. (**C**) Flow, film thickness, and setting time were measured immediately after heat treatment; pH and calcium ion release were evaluated after 24 h cure at 37 ± 1 °C with 95% relative humidity; and surface analysis, FT-IT, and TGA were evaluated after 48 h set under the same conditions.

The procedures for heat application on ERBS (e.g., AH Plus) and CSBS (e.g., BioRoot Flow, BioRoot RCS, and AH Plus Bioceramic) were conducted as described previously (Figure 1) [20]. Before heat application, each sealer was placed in a 2 mL plastic tube (Safe-Lock Tubes, 1.5 mL; Eppendorf, Hamburg, Germany). In accordance with the manufacturer’s recommendations, BioRoot RCS was mixed on a glass plate and subsequently transferred into a 2 mL plastic tube. On the other hand, BioRoot Flow and AH Plus Bioceramic were directly dispensed from syringes. AH Plus was transferred into a 2 mL plastic tube using a mixing tip attached to the syringe entrance. For heat application, the tubes were immersed in a water bath (hot plate stirrer, MSH-20D; DAIHAN Scientific, Wonju, Republic of Korea). The K-type thermocouple (version 1.27; GHM Messtechnik, Hennigsdorf, Germany) was placed in the tubes to adjust the temperatures of the sealers to 37 °C and 100 °C. Following that, the samples were maintained at these temperatures for 30 s, after which they were cooled to 37 °C in a separate temperature-adjusted water tank. Six samples were prepared for each experimental group.

### 2.2. Physical and Chemical Properties

#### 2.2.1. Flow

Given that previous studies [20,22] revealed increased sealer viscosity after heat treatment, the flow of the sealers was tested with slight modifications to the ISO standard [23]. Weights of 0.05 mL for each sealer were measured: 0.140 g for AH Plus; 0.1285 g for AH Plus Bioceramic; 0.110 g for BioRoot RCS; and 0.114 g for BioRoot Flow. The samples were placed on a glass plate (40 mm × 40 mm × 5 mm) using a graduated 1 mL syringe (BD Luer-Lok 1 mL Syringe; Becton Dickinson and Co., Franklin Lakes, NJ, USA). Another glass plate was placed on top after 3 min and then pressurized with a 100 g weight in the center (*n* = 6/group). The weight was removed after 10 min to determine the maximum and minimum diameters of the samples using a digital caliper (CD-P15S; Mitutoyo Corp., Kanagawa, Japan); in cases of difference > 1 mm, the diameters were re-evaluated.

#### 2.2.2. Film Thickness

In accordance with ISO 6876:2012 [23], the film thickness was evaluated by placing a 0.05 mL sealer between glass plates (200 ± 25 mm^2^). At 3 min after mixing the sealers, a 150 N load was applied with a universal testing machine (ZP-500N; Imada, Toyohashi, Japan) to ensure that the sample filled the area between the glass plates completely. Ten minutes after mixing, the thicknesses of the two glass plates and the sample were measured using a digital micrometer (293 Series; Mitutoyo Corp.) (*n* = 6/group). The film thickness was calculated as the difference between the thicknesses of samples with and without glass plates.

#### 2.2.3. Setting Time

The setting time was evaluated in accordance with ISO 6876:2012 [23]. Hydraulic sealers (e.g., BioRoot Flow, BioRoot RCS, and AH Plus Bioceramic) that require moisture for setting were filled in pre-treated gypsum molds (10 mm in diameter and 1 mm in height) and maintained at 95% relative humidity and 37 ± 1 °C for 24 h before the measurement. A stainless-steel ring (10 mm in diameter and 2 mm in height) was used for AH Plus, which does not require moisture. All sealers were placed in a cabinet with 95% relative humidity at 37 ± 1 °C. Furthermore, an indenter (100 ± 0.5 g) with a 2.0 ± 0.1 mm diameter tip to obtain repeated measurements until no indentation was formed (*n* = 6/group).

#### 2.2.4. Calcium Ion Release

The sealers were filled in Teflon molds (7.75 mm in diameter and 1.5 mm in height) and then stored for 24 h in an incubator with 95% relative humidity and 37 ± 1 °C. The weights of samples were measured on a scale with a precision of 0.001 g and placed in glass vials with 7.5 mL Hank’s Balanced Salt Solution (HBSS; H8264—500 mL; Sigma-Aldrich, St. Louis, MO, USA). Then, the solution was incubated (95% relative humidity, 37 ± 1 °C) for 7 days (*n* = 6/group). Finally, calcium ion release was measured using an inductively coupled plasma optical emission spectrometer (ICP-OES, iCAP 6000 Series; Thermo Fisher Scientific, Waltham, MA, USA).

#### 2.2.5. Solubility

The sealers were filled in Teflon molds (7.75 mm in diameter and 1.5 mm in height) with a nylon thread attached to the surface. Then, cellophane film and glass plates were placed on top before setting samples in an incubator (311-TIF; Thermo Fisher Scientific) with 95% relative humidity at 37 ± 1 °C for 48 h. The samples were dried for 24 h before the weight was measured using a precision electronic scale (FX-200i; AND A&D Corp., Tokyo, Japan). Next, the samples were placed in a glass vial containing 7.5 mL phosphate-buffered saline (PBS-001–500; SolBioPharm Corp., Suwon, Republic of Korea) and then stored for 24 h or 28 days in an incubator (37 ± 1 °C, 95% relative humidity) (*n* = 6/group). Later, samples were dried for a further 24 h to measure the mass loss rate compared to the initial mass.

#### 2.2.6. pH Changes

Polyethylene tubes (1.5 mm in internal diameter and 10 mm in length) were filled with sealers and incubated for 24 h at 95% relative humidity and 37 ± 1 °C. Then, the samples were placed in glass vials containing 10 mL HBSS and stored in an incubator (95% relative humidity, 37 ± 1 °C). Next, the pH values were measured at 7-day intervals (1, 7, 14, 21, and 28 days) using a pH meter (Orion Star A211; Thermo Fisher Scientific) (*n* = 6/group).

#### 2.2.7. Thermogravimetric Analysis (TGA)

Teflon molds (7.75 mm in diameter and 1.5 mm in height) were filled with sealers and incubated at 95% relative humidity and 37 ± 1 °C (n = 2/group). Then, the samples were placed in a platinum pan, and a TGA (TGA Q 50; TA Instruments, New Castle, DE, USA) with a built-in high-sensitivity scale was used to measure 6–8 mg of the samples. The weight changes were monitored during heat treatment at 10 °C/min in a nitrogen gas environment, which increased the temperature from room temperature (RT) to 200 °C.

#### 2.2.8. Fourier-Transform Infrared Spectroscopy (FT-IR)

**Figure 2:**
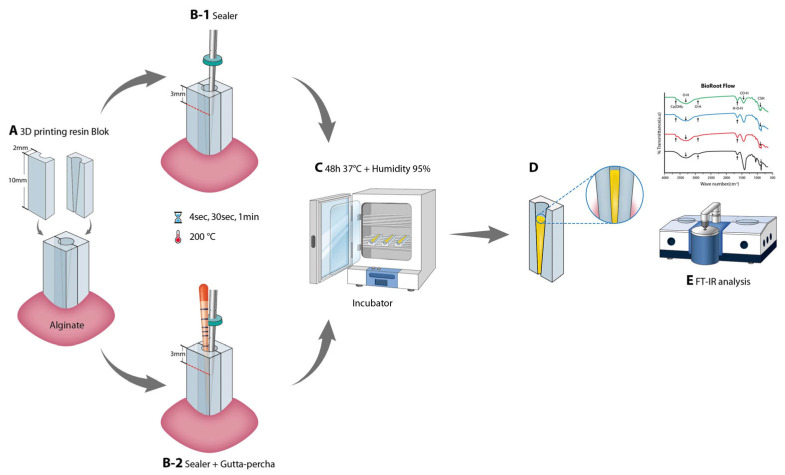
Schematic representation of FT-IR process. (**A**) Preparing a resin-block sample; (**B1**,**B2**) sealer and gutta-percha were inserted in the resin block and the tip was inserted to a depth of 3 mm from the top; (**C**) samples set for 48 h in an incubator with 95% relative humidity at 37 ± 1 °C; (**D**) FT-IR analysis.

FT-IR is typically conducted by placing sealers in an oven and adjusting the external temperature to 100 °C [24,25]. However, to simulate the clinical environment, we used three-dimensional printed root-canal models (10 mm in height with a taper of 0.2 mm). For the experiment, the root-canal models were manufactured separately for later removal (Figure A), and the heat was directly applied to the sealers in the root canal using a System B heat source (Analytic Technology, Redmond, WA, USA). In one group, only the sealer was applied to the root-canal model, whereas in the second group, both the sealer and gutta-percha (Easyinsmile, Staten Island, NY, USA) were applied. In accordance with the manufacturer’s instructions, the sealer was mixed before placement, and the gutta-percha cone (#30 with a taper of 0.06 mm, cut to a length of 20 mm) was then slightly coated with sealer mixture and slowly inserted into the root-canal model (Figure 2B). System B was set to 200 °C, and the tip was inserted into the root canal to a depth of 3 mm under continuous heating. The pressure was maintained for 4 s, 30 s, and 1 min. A 0.08 mm tip was used in System B. The samples were cured in an incubator with 95% relative humidity and at 37 ± 1 °C for 48 h (Figure 2C). Using the FT-IR (Vertex-70V; Bruker, Karlsruhe, Germany) in an attenuated total reflectance mode with a spectral resolution of 4 cm^−1^, the spectrum of the solid component was evaluated with a range of 500–4000 cm^−1^ in vacuum (Figure 2D).

### 2.3. Surface Characterization

#### Field Emission Scanning Electron Microscope

The sealers were filled in Teflon molds (7.75 mm in diameter and 1.5 mm in height) (*n* = 2/group) and then placed in an incubator (95% relative humidity, 37 ± 1 °C) for 48 h. The samples were stored in HBSS for 7 days before drying in a desiccator (VDR-20; JEIO TECH, Daejeon, Republic of Korea) for 24 h. The samples were analyzed using a field emission scanning electron microscope (Apreo S Hivac; FEI company, Hillsboro, OR, USA) with an energy-dispersive X-ray (EDX) spectrometer after carbon coating. A standard electron microscope (SEM) image was obtained at a magnification of 3000×. The elemental composition of each material was quantified using EDX analysis.

### 2.4. Statistical Analysis

Statistical analyses were performed using SPSS software (version 29.0; IBM Corp., Armonk, NY, USA). Paired *t*-tests were used to analyze the flow, film thickness, setting time, solubility, and calcium ion release of each sealer according to the temperature. One-way analysis of variance and Tukey’s post hoc tests were used to compare the properties of each sealer type. Repeated measures analysis of variance was performed to determine pH changes over time. *p*-values < 0.05 were considered indicative of statistical significance.

## 3. Results

### 3.1. Physicochemical Properties

**Figure 3:**
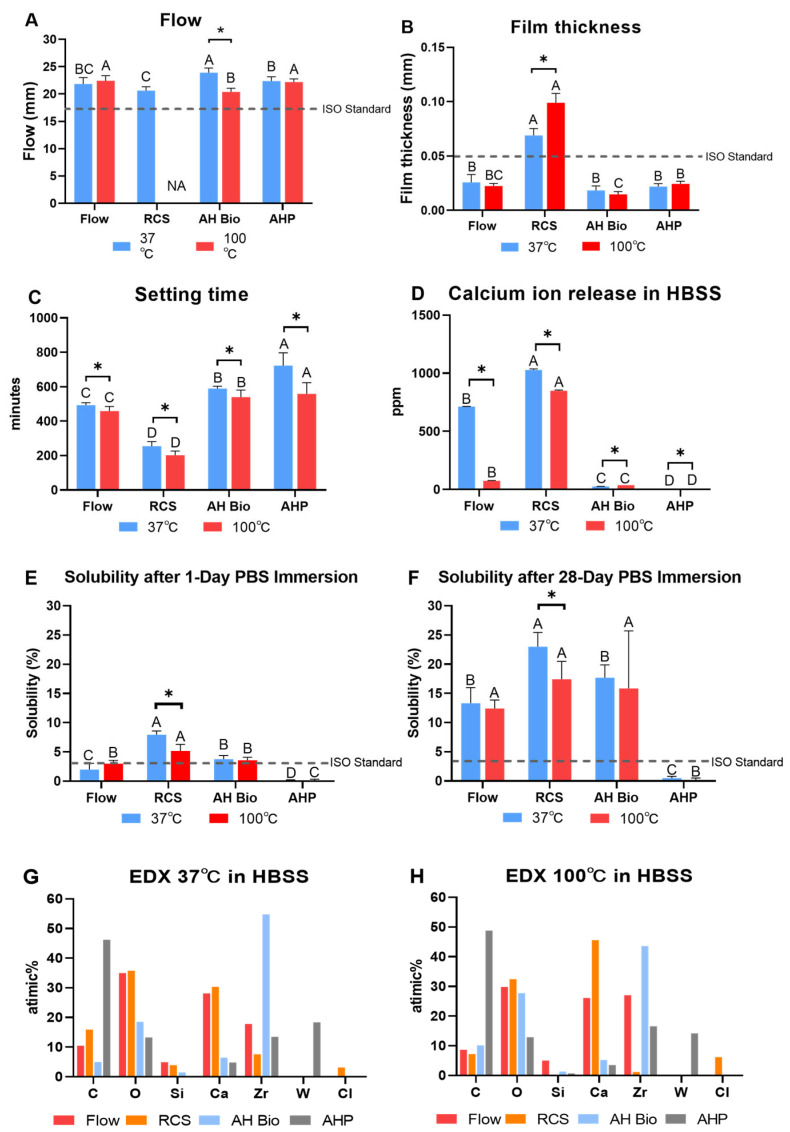
Flow, film thickness, setting time, calcium ion release, solubility, and EDX. (**A**) The flow at 37 °C and 100 °C. (**B**) The film thickness at 37 °C and 100 °C. (**C**) The setting time at 37 °C and 100 °C. (**D**) The calcium ion released at 37 °C and 100 °C. (**E**) The solubility after 1 day of immersion in PBS at 37 °C and 100 °C. (**F**) The solubility after 1 day of immersion in PBS at 37 °C and 100 °C. (**G**) EDS at 37 °C in HBSS. (**H**) EDS at 100 °C in HBSS. Different uppercase letters represent significant differences for the other sealers at the same experimental temperature (*p* < 0.05, A > B > C > D based on mean value). The *dashed line* represents ISO 6876 cutoff levels. * *p* < 0.05 (analysis of variance with Tukey’s multiple comparison test, paired *t* test).

Figure 3 presents the physicochemical properties of four sealers at different temperatures.

#### 3.1.1. Flow

The flow of AH Plus Bioceramic was significantly reduced when 100 °C heat was applied (*p* < 0.05). The flow of BioRoot RCS could not be determined according to the ISO standard, as it was partially set by applying 100 °C heat. No significant differences were observed in the flow between BioRoot Flow and AH Plus at different temperatures (*p* > 0.05). BioRoot Flow, AH Plus Bioceramic, and AH Plus complied with the ISO standard (>17 mm) at 37 °C and 100 °C.

#### 3.1.2. Film Thickness

BioRoot RCS exhibited significantly increased film thickness when 100 °C heat was applied (*p* < 0.05). However, no significant differences were observed in the film thickness of BioRoot Flow, AH Plus Bioceramic, and AH Plus at different temperatures (*p* > 0.05).

#### 3.1.3. Setting Time

All sealers showed significantly decreased setting time when 100 °C heat was applied (*p* < 0.05). At 37 °C and 100 °C, AH Plus exhibited the longest setting time, followed by AH Plus Bioceramic, BioRoot Flow, and BioRoot RCS (*p* < 0.05).

#### 3.1.4. Calcium Ion Release

BioRoot Flow and BioRoot RCS showed a significant decrease in calcium ion release at 100 °C (*p* < 0.05). At 37 °C and 100 °C, BioRoot RCS exhibited the highest calcium ion release, followed by BioRoot Flow, AH Plus Bioceramic, and AH Plus, with significant differences among the sealers (*p* < 0.05).

#### 3.1.5. Solubility

BioRoot RCS exhibited significantly reduced solubility at 100 °C after 24 h and 28 days (*p* < 0.05). However, BioRoot Flow, AH Plus Bioceramic, and AH Plus did not demonstrate any significant differences in solubility among various temperatures (*p* > 0.05). The solubility of BioRoot Flow, BioRoot RCS, and AH Plus Bioceramic exceeded the ISO standard (<3%), whereas AH Plus complied with the ISO standard.

#### 3.1.6. Changes in pH 

Changes in pH were evaluated at 37 °C and 100 °C using the HBSS solution (Table 1). All sealers except AH Plus had an alkaline pH, regardless of the temperature. Furthermore, compared to AH Plus, all sealers exhibited significantly higher pH values by day 28 (*p* < 0.05). Those three also exhibited significantly reduced pH values at 100 °C by day 28. However, AH Plus demonstrated no significant changes in pH over time at 37 °C or 100 °C (*p* > 0.05).

#### 3.1.7. TGA

**Figure 4:**
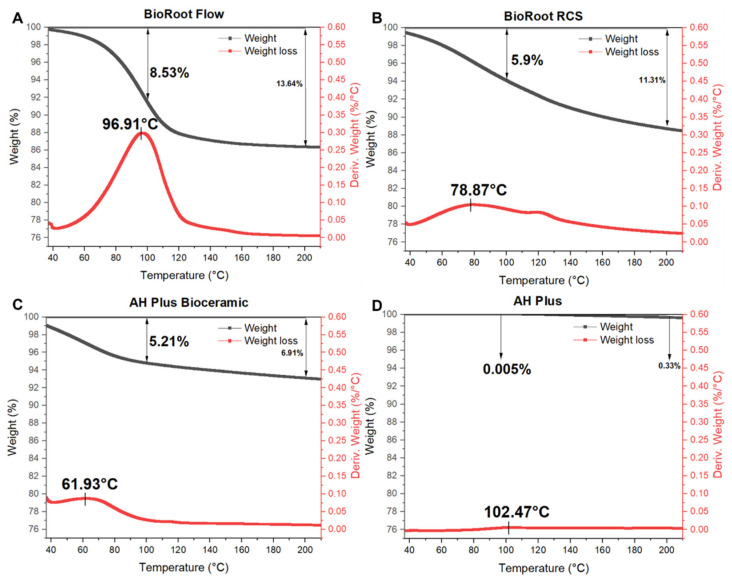
Weight loss (black line) and temperature at which weight loss begins (red line) for four sealers analyzed using TGA. (**A**) BioRoot Flow, (**B**) BioRoot RCS, (**C**) AH Plus Bioceramic, (**D**) AH Plus.

Figure 4 presents the changes in the weights of the sealers at a high temperature determined via TGA. All sealers exhibited increasing weight loss until 200 °C followed by stabilization. TGA was performed for each group, with the temperature increasing from RT to 200 °C. At 100 °C, the residual masses of sealers were 91.47% for BioRoot Flow, 94.1% for BioRoot RCS, 94.79% for AH Plus Bioceramic, and 99.93% for AH Plus. Furthermore, weight loss started for these sealers at 96.91 °C, 78.87 °C, 61.93 °C, and 102.47 °C, respectively (Figure 4).

#### 3.1.8. FT-IR

**Figure 5:**
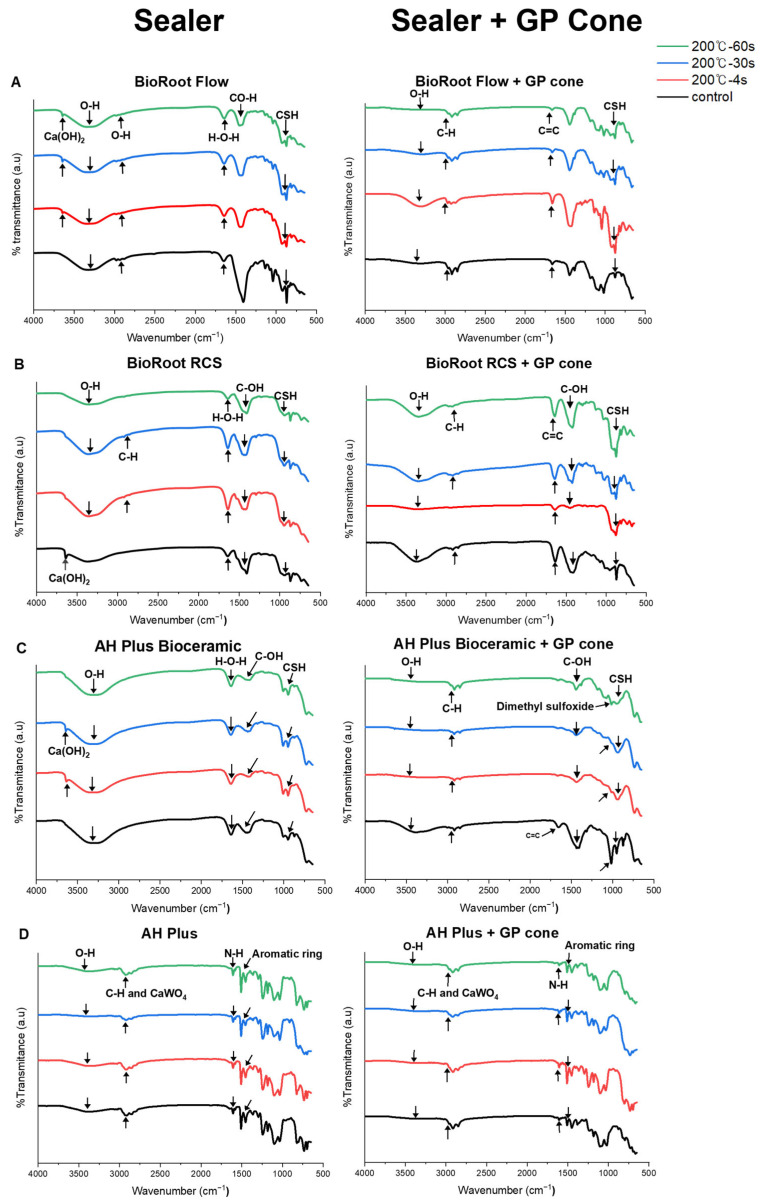
FT-IR spectra of sealers with and without gutta-percha at 37 °C (black) and 200 °C for 4 s (red), 30 s (blue), and 60 s (green). (**A**) BioRoot Flow, (**B**) BioRoot RCS, (**C**) AH Plus Bioceramic, (**D**) AH Plus. All the sealers showed a water O-H absorption band at 3400 cm^−1^. BioRoot RCS, when heated to 100 °C, Ca(OH)_2_ spectrum observed at 3646 cm^−1^ disappeared, the CSBS (**A**–**C**) showed a small Ca(OH)_2_ peak at 3646 cm^−1^, and a CSH spectrum from 970 to 1000 cm^−1^. AH Plus (**D**) exhibited N-H stretching vibration at 1607 cm^−1^. No chemical changes were detected in all sealers when mixed with gutta-percha.

Figure 5 presents the changes in the chemical structure of sealers using FT-IR after heat application using system B for 4 s, 30 s, and 1 min. No changes in chemical structure were observed among the sealers after heat application. Furthermore, all sealers revealed the presence of O-H (water) at 3400 cm^−1^ and 1650 cm^−1^. The CSBS group exhibited a small Ca(OH)_2_ peak at 3646 cm^−1^ and a calcium silicate hydrate spectrum at 970–1000 cm^−1^. For AH Plus Bioceramic and BioRoot RCS, the Ca(OH)_2_ peak disappeared at 3646 cm^−1^ when 200 °C heat was applied. AH Plus demonstrated N-H stretching vibration at 1607 cm^−1^. FT-IR analysis of the gutta-percha and sealer mixtures revealed minimal chemical changes between RT and 200 °C, confirming that the gutta-percha did not affect the sealer at a high temperature. 

### 3.2. Field Emission Scanning Electron Microscope

**Figure 6:**
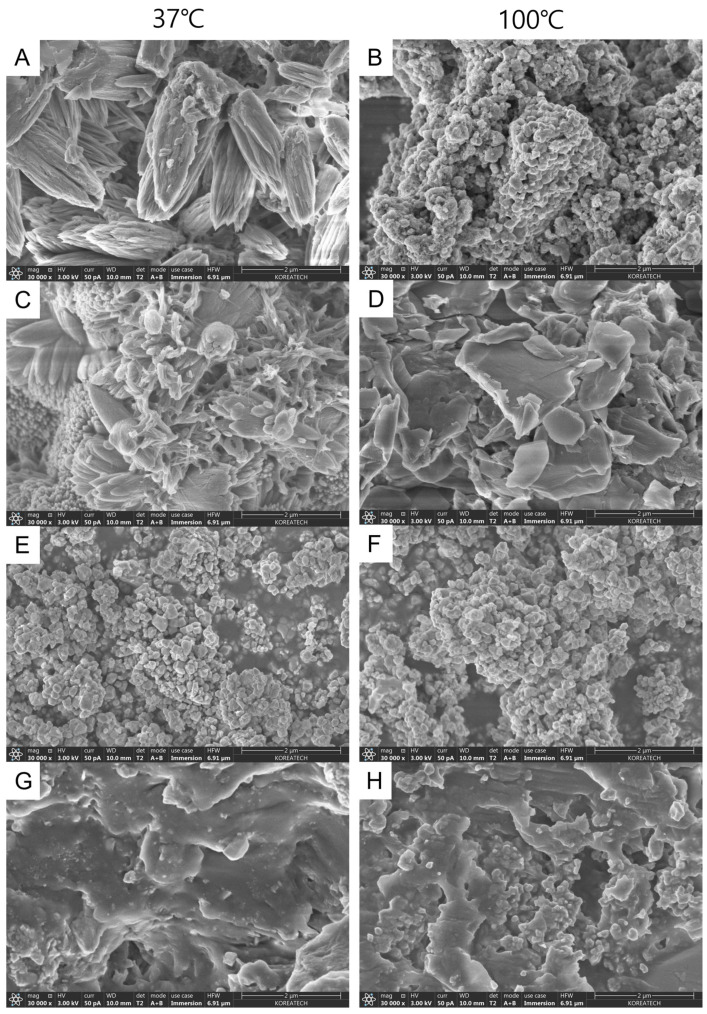
Scanning electron microscopic image of (**A**) BioRoot Flow 37 °C, (**B**) BioRoot Flow 100 °C, (**C**) BioRoot RCS 37 °C, (**D**) BioRoot RCS 100 °C, (**E**) AH Plus Bioceramic 37 °C, (**F**) AH Plus Bioceramic 100 °C, (**G**) AH Plus 37 °C, and (**H**) AH Plus 100 °C in HBSS (30,000×).

Figure 6 presents the surface analysis of four sealers according to the temperature. Needle-like precipitates were observed in BioRoot Flow at 37 °C (Figure 6A), while precipitates consisting of round particles were observed at 100 °C (Figure 6B).

In BioRoot RCS, the precipitates were round and elongated at 37 °C, which changed to an irregular crystalline structure at 100 °C (Figure 6C,D). AH Plus Bioceramic demonstrated round particles before and after heat application (Figure 6E,F). AH Plus exhibited a relatively smooth surface, with a preserved regular matrix after heat application (Figure 6G,H). A sharp precipitate was observed in BioRoot Flow and BioRoot RCS (CSBS), indicating that the precipitate was formed due to the interaction between the sealer and the surrounding environment [26]. An EDX analysis revealed that the main components of CSBS (BioRoot Flow, BioRoot RCS, and AH Plus Bioceramic) were calcium, silicon, and zirconium, a radiopaque agent. Furthermore, BioRoot RCS contained a setting accelerator-chlorine peak. The main components of AH Plus were zirconium, tungsten, silicon, and calcium (Figure 3G,H). 

## 4. Discussion

We compared the physicochemical and surface properties of three CSBSs (BioRoot Flow, BioRoot RCS, and AH Plus Bioceramic) and ERBS (AH Plus) at a high temperature to evaluate the suitability of CSBS use during sealer-based and warm obturation techniques. The clinical performance of endodontic sealers is related to their physicochemical and biological properties. Therefore, an understanding of these characteristics provides useful information for selecting the most suitable sealing material [8]. With the development of new endodontic sealers, clinicians should select the most appropriate sealer based on the physicochemical properties. To the best of our knowledge, this is the first study to compare the characteristics of BioRoot Flow, based on the optimal properties suggested by Grossman et al. [2]. and the requirements for endodontic sealers specified in ISO 6876:2012 [23]. Previous studies have investigated the chemical changes in sealers when subjected to heat in an oven using FT-IR analysis [24,27] but not during thermal application using clinically used heat sources, such as system B.

Flow and film thickness are crucial physical properties that determine the sealing ability of sealers used for filling the complex root-canal structure [28,29]. BioRoot Flow, AH Plus Bioceramic, and AH Plus complied with ISO standards in terms of flow and film thickness before and after heat application. However, BioRoot RCS exhibited increased film thickness at 100 °C. These results are in line with those of previous studies that have compared the properties of various sealers at different temperatures [20].

A prolonged setting time may result in unfavorable outcomes due to sealer dissolution on making contact with oral-tissue fluids [24,30,31]. In this study, the setting times of all sealers were significantly reduced after heat treatment, probably due to moisture evaporation (solidifying the sealer) or shortened primary setting time [20]. In particular, BioRoot RCS exhibited the shortest setting time, possibly due to exposure of calcium chloride to high temperature which shortened the setting time [24,32]. 

Calcium ions enhance bioactivity and promote root canal and periodontal tissue regeneration [33]. In the present study, calcium release was significantly reduced in BioRoot Flow and BioRoot RCS at 100 °C. These results may be attributable to a chemical reaction between calcium ions and water, which is enhanced by heat application and leads to water evaporation [34]. Furthermore, BioRoot RCS consistently demonstrated the highest calcium ion release at all temperatures, which is in line with previous studies that have compared the calcium release from various calcium silicate-based sealers [7,24]. According to the manufacturer, AH Plus Bioceramic contains 5–15 wt% tricalcium silicate [11], whereas the manufacturers of BioRoot Flow and BioRoot RCS have not specified the tricalcium content. BioRoot Flow contains calcium hydroxide and calcium carbonate in addition to tricalcium silicate [12]. BioRoot RCS, which includes calcium chloride, exhibited greater biocompatibility than the other CSBS products [7,24]. The differences in calcium content suggest that BioRoot RCS and BioRoot Flow release more calcium ions than AH Plus Bioceramic. 

The obturation quality of root-canal sealers is crucial for preventing microbial growth [35]. In this study, the solubility of the CSBS group exceeded the ISO standards both before and after heat application, whereas AH Plus fulfilled the standards under both conditions. CSBS exhibited higher solubility than ERBS, which is in line with previous studies [7,36,37]. However, our results are not in line with those of another study that demonstrated no significant differences before and after heat application [24]. Notably, these discrepancies in the solubility between the present and previous studies may be attributable to differences in experimental conditions and sample drying methods [36]. A previous study revealed that the presence of water-insoluble tungsten in AH Plus can affect its solubility [38]. In addition, we found that the tungsten included in AH Plus can explain the difference in solubility, as observed in SEM images, between AH Plus and CSBS. However, the high solubility of BioRoot RCS observed in the present study can be attributed to the irregular porous surface observed in the SEM images [31,38]. Previous studies have suggested that a greater size of hydrophilic particles (nm) is associated with a larger surface area, allowing greater contact of liquid molecules with the sealer during immersion, leading to enhanced solubility [31,38]. In addition, in this study, BioRoot RCS exhibited the highest calcium ion release, leading to its high solubility due to the release of initial hydroxide and calcium ions [39].

Previous studies have demonstrated that sealers with an alkaline pH have antibacterial effects, osteogenic ability, and biocompatibility [40,41]. In the present study, the pH was higher for CSBS than AH Plus, which is consistent with the results of other studies [24]. The pH was significantly reduced after heat application of BioRoot Flow, BioRoot RCS, and AH Plus Bioceramic. After heat application, the calcium hydroxide produced can influence the numbers of ions released [34], and CSBS affects the pH due to OH-evaporation. However, further studies are needed to evaluate the interaction between pH in sealers and biological conditions. In particular, additional studies on biocompatibility with surrounding tissues, bone formation, and antibacterial activity according to pH changes in sealers are needed.

The weight stability of the sealer at a high temperature, as assessed using TGA, was similar to that observed in previous studies [27], with higher weight loss observed for CSBS than for AH Plus. These results vary depending on the components and moisture content of the sealers [42]. Therefore, moisture loss after heat application may have contributed to our study results. The greater weight loss in BioRoot Flow compared to pre-mixed AH Plus Bioceramic may be attributable to its higher calcium oxide content [39].

According to the FT-IR analysis, the chemical structure of the sealers was unchanged within the temperature range used in this study. Furthermore, the chemical structure was unaffected by heat in the gutta-percha and sealer mixture. Previous studies have demonstrated that the N-H bond is decomposed in AH Plus with a temperature over 100 °C, causing irreversible changes [16,17,18]. However, no N-H stretching vibration was observed in the present study. This discrepancy may be due to the higher temperature ranges (140–250 °C) or longer time (1–10 min) used in previous studies [17,18]. Furthermore, calcium silicate hydrate was formed in BioRoot Flow, BioRoot RCS, and AH Plus Bioceramic at 970–1000 cm^−1^, regardless of heat application, confirming the high-temperature resistance of CSBS within the temperature range used in this study, which is in line with previous studies [27].

In previous studies, sealers were heated in an oven for 1–10 min to evaluate their physicochemical properties before and after heating [24]. However, this testing method promotes the evaporation of certain components. The heat-application method used in the present study was adopted from previous studies [20,22]. To simulate a clinical environment, the samples were enclosed in plastic tubes to maintain a seal. In a previous study [17,18], system B was set to a temperature of 200 °C and operated for 30 s to 2 min, resulting in the temperature of the plugger being 50–126 °C [17,18]. In the present study, a temperature of 37 °C was used to simulate the human body temperature, with the system B tip temperature set at 100 °C for 30 s. Furthermore, to simulate the temperature decrease that occurs within the human body, the samples initially maintained at 100 °C were gradually cooled down to 37 °C.

The present study had several limitations. First, although the temperatures used in this study were set to simulate clinically relevant conditions, it proved challenging to precisely replicate the temperatures encountered in an actual clinical setting. Therefore, further studies under clinical conditions are required. Second, the evaluation period for solubility was 28 days. However, a long observation period is needed to evaluate the long-term material stability and structural changes in root-canal sealers. Third, the BioRoot RCS was manually mixed by the operator, which introduces an operator-dependent factor that may affect the outcomes. 

These limitations should be considered when interpreting our results for the clinical application of sealers. Our results should help facilitate the development of materials with improved function and stability at high temperatures.

## 5. Conclusions

BioRoot Flow, AH Plus Bioceramic, and AH Plus have stable physicochemical properties, which makes them suitable for clinical application under thermal conditions. Although BioRoot RCS did not exhibit an altered chemical structure at high temperatures, the film thickness was increased and the pH and solubility were decreased after heat application. Furthermore, all sealers demonstrated a reduction in calcium ion release post-heat application. Therefore, when applying BioRoot RCS under thermal conditions, it is important to maintain an understanding of the alterations in its physical properties and be cautious in its application, such as during the warm obturation technique.

## Figures and Tables

**Table 1:** Change in pH of sealers after immersion in HBSS for 1, 7, 14, 21, and 28 days at each experimental temperature.

Material	1 Day		7 Day		14 Day		21 Day		28 Day		Time x Group
	37 °C	100 °C	37 °C	100 °C	37 °C	100 °C	37 °C	100 °C	37 °C	100 °C	
Flow	10.61 ± 0.09 ^a^	10.71 ± 0.09 ^a^	10.53 ± 0.17 ^a^	10.26 ± 0.17 ^a^	10.45 ± 0.07 ^b^	11.12 ± 0.07 ^a^	10.01 ± 0.23 ^a^	9.02 ± 0.23 ^b^	9.64 ± 0.03 ^a^	8.48 ± 0.03 ^b^	<0.05 *
RCS	11.24 ± 0.04 ^a^	10.34 ± 0.04 ^b^	9.86 ± 0.08 ^a^	9.64 ± 0.08 ^a^	10.66 ± 0.14 ^b^	10.02 ± 0.14 ^a^	10.17 ± 0.14 ^b^	8.54 ± 0.14 ^a^	9.65 ± 0.04 ^a^	8.07 ± 0.04 ^b^	<0.05 *
AH Bio	9.86 ± 0.05 ^a^	9.41 ± 0.05 ^b^	9.41 ± 0.19 ^a^	8.61 ± 0.19 ^b^	9.56 ± 0.03 ^b^	9.63 ± 0.03 ^a^	8.61 ± 0.20 ^a^	7.79 ± 0.20 ^b^	8.6 ± 0.12 ^a^	7.74 ± 0.12 ^b^	<0.05 *
AH Plus	7.81 ± 0.14 ^a^	7.22 ± 0.14 ^b^	7.84 ± 0.06 ^a^	7.45 ± 0.06 ^b^	7.67 ± 0.1 ^a^	7.50 ± 0.1 ^a^	7.59 ± 0.07 ^a^	7.53 ± 0.07 ^a^	7.38 ± 0.14 ^a^	7.62 ± 0.14 ^a^	>0.05 *

Values are expressed as mean ± SE (*n* = 6 for each group). Analysis of variance for repeated measurements (RM ANOVA) and Bonferroni correction for multiple comparisons were used to examine differences between time points. If the time x intervention interaction was significant, RM-ANOVA models were calculated with stratification by intervention or control group. If the assumption of sphericity was violated (*p* < 0.05, Mauchly’s test of sphericity “*”), the Greenhouse–Geisser correction was used to estimate *p* values. Flow, BioRoot Flow; RCS, BioRoot RCS; AH Bio, AH Plus Bioceramic. Different lowercase letters indicate significant differences between the sealers within each temperature (*p* < 0.05, a > b based on mean value).

## Data Availability

The datasets used and/or analyzed during this study are available from the corresponding author on reasonable request.
